# Combination with l-Menthol Enhances Transdermal Penetration of Indomethacin Solid Nanoparticles

**DOI:** 10.3390/ijms20153644

**Published:** 2019-07-25

**Authors:** Noriaki Nagai, Fumihiko Ogata, Mizuki Yamaguchi, Yuya Fukuoka, Hiroko Otake, Yosuke Nakazawa, Naohito Kawasaki

**Affiliations:** 1 Faculty of Pharmacy, Kindai University, 3-4-1 Kowakae, Higashi-Osaka, Osaka 577-8502, Japan; 2 Faculty of Pharmacy, Keio University, 1-5-30 Shibakoen, Minato-ku, Tokyo 105-8512, Japan

**Keywords:** indomethacin, l-menthol, nanoparticle, skin, drug delivery

## Abstract

This study designed the transdermal formulations containing indomethacin (IMC)—1% IMC was crushed with 0.5% methylcellulose and 5% 2-hydroxypropyl-β-cyclodextrin by the bead mill method, and the milled IMC was gelled with or without 2% l-menthol (a permeation enhancer) by Carbopol^®^ 934 (without menthol, N-IMC gel; with menthol, N-IMC/MT gel). In addition, the drug release, skin penetration and percutaneous absorption of the N-IMC/MT gel were investigated. The particle sizes of N-IMC gel were approximately 50–200 nm, and the combination with l-menthol did not affect the particle characterization of the transdermal formulations. In an in vitro experiment using a Franz diffusion cell, the skin penetration in N-IMC/MT gel was enhanced than the N-IMC gel, and the percutaneous absorption (*AUC*) from the N-IMC/MT gel was 2-fold higher than the N-IMC gel. On the other hand, the skin penetration from the N-IMC/MT gel was remarkably attenuated at a 4 °C condition, a temperature that inhibits all energy-dependent endocytosis. In conclusion, this study designed transdermal formulations containing IMC solid nanoparticles and l-menthol, and found that the combination with l-menthol enhanced the skin penetration of the IMC solid nanoparticles. In addition, the energy-dependency of the skin penetration of IMC solid nanoparticles was demonstrated. These findings suggest the utility of a transdermal drug delivery system to provide the easy application of solid nanoparticles (SNPs).

## 1. Introduction

Indomethacin (IMC) is a non-steroidal anti-inflammatory drug (NSAIDs) that is widely prescribed as therapy for inflammation, fever and pain. The Biopharmaceutical Classification System (BCS) lists indomethacin as a Class II drug, and the pK and logP of IMC are 4.5, 2.2, respectively. The IMC acts by blocking cyclooxygenase (COX), which is involved in the synthesis of prostaglandins from arachidonic acid. However, the inhibition of COX by the oral administration of IMC decreases defense functions in the gastrointestinal system and is associated with undesirable side effects involving gastroduodenal mucosal injury [1,2,3]. In addition, it has been reported that the decreased defense function increased the direct stimulation by IMC in the gastrointestinal system, and that this stimulation was also related to the onset of gastroduodenal mucosal injury [4,5]. One well-known method to avoid the problems associated with these side effects involves transdermal drug delivery (TDD).

Skin can be utilized as a route for drug delivery, and TDD offers many advantages over oral administration, such as the avoidance of gastroduodenal mucosal injury and the first pass effect. In addition, TDD provides for more control of plasma drug levels, low enzyme-dependent degradation, and reduces the frequency of drug application [6]. On the other hand, the main disadvantage of TDD concerns the low permeability of skin, which limits drug penetration, and the major challenge of TDD is overcoming the stratum corneum (SC), which is a main barrier for drugs. Many studies have designed various methods to enhance TDD, such as drug incorporation into liposomes, incorporation of penetration enhancers, microneedles, transdermal patches, and microemulsions [7,8,9,10,11]. In particular, nanotechnology is an evolving trend in TDD, and includes several forms, such as solid nanoparticles (SNPs), dendrimers, liposomes, lipid nanocarriers, polymeric nanoparticles, nanocrystals and nanoemulsions. These small size drugs allow them to adhere to the SC and enhance the drug penetration into the deeper layers of the skin, resulting in increased drug absorption [12,13,14]. The authors also reported that SNPs of NSAIDs (IMC and ketoprofen) prepared by the mill treatment showed high rates of percutaneous absorption, and that energy-dependent endocytosis was related to the mechanism of skin penetration [15,16]. In addition, the therapeutic effect on inflammation of transdermal formulations containing SNPs of ketoprofen and tranilast is greater as compared with traditional formulations (dissolution type, commercially available traditional formulations) [15,16,17]. Furthermore, the authors found that over 100 nm-SNPs (>100 nm) were inhibited in the SC, and only less than 100 nm SNPs (<100 nm) can penetrate into the skin tissue via SC [15,16,17,18,19]. Therefore, the achievement of SC-penetration by over 100 nm-SNPs may enhance the practical application of SNPs-based TDD.

Due to the excellent barrier function of the SC, the need for safe and effective enhancers to improve the transdermal absorption of drugs is well recognized [20]. Further, l-menthol has been shown to increase skin absorption by altering the barrier properties of the SC [21], and has acted as an enhancer of drug skin permeation. This study prepared transdermal formulations containing IMC SNPs (N-IMC gel) and transdermal formulations containing IMC SNPs and l-menthol (N-IMC/MT gel), and evaluated the stability of the N-IMC/MT gel. Moreover, the drug release, skin penetration and percutaneous absorption of the N-IMC/MT gel were also investigated.

## 2. Results

### 2.1. Evaluation of Transdermal Formulations Containing IMC SNPs and l-Menthol

**Figure 1:**
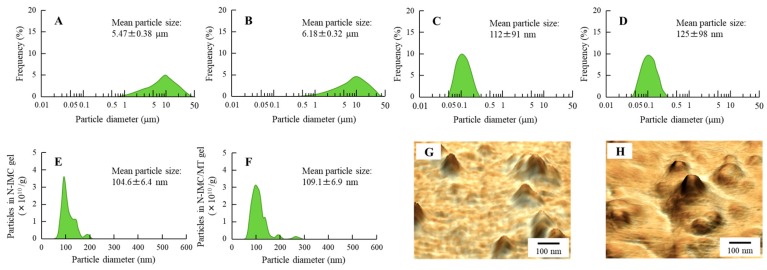
Particle analysis of N-IMC and N-IMC/MT gels immediately after the bead mill treatment. (**A**) and (**B**); the particle size frequencies in transdermal formulations containing indomethacin (IMC) powder (P-IMC, **A**) and transdermal formulations containing IMC powder and l-menthol (P-IMC/MT, **B**) gels by SALD-7100. (**C** and **D**); the particle size frequencies of N-IMC (**C**) and N-IMC/MT (**D**) gels by SALD-7100. (**E** and **F**); the particle size frequencies of N-IMC (**E**) and N-IMC/MT (**F**) gels by NANOSIGHT LM10. (**G** and **H**); atomic force microscope (AFM) images of N-IMC (**G**) and N-IMC/MT (**H**) gels by SPM-9700. The particle size of IMC was decreased to approximately 50–200 nm, and no differences were observed in the particle characterizations between the N-IMC and N-IMC/MT gels.

**Figure 2:**
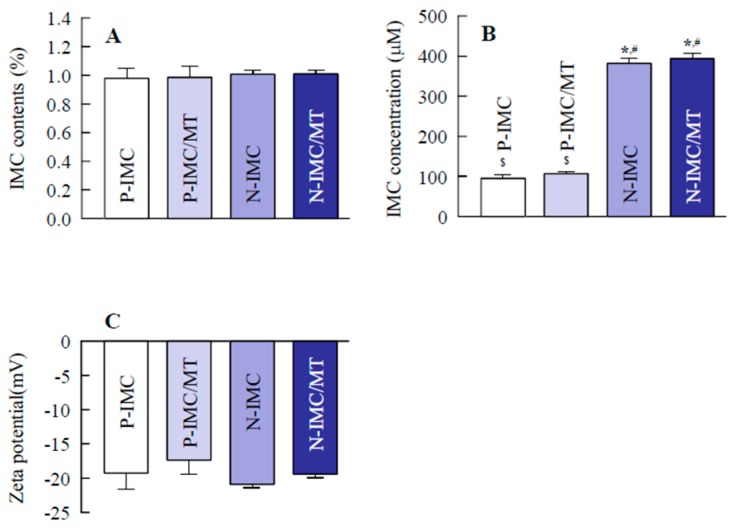
The stability and particle characterization of N-IMC and N-IMC/MT gels. (**A**); the dispersity in the base of IMC transdermal formulations. (**B**); the solubility of IMC in transdermal formulations. (**C**); the zeta potential of IMC in transdermal formulations. *n* = 6–8. * *p* < 0.05 vs. P-IMC gel for each category. ^#^ *p* < 0.05 vs. P-IMC/MT gel for each category. ^$^ *p* < 0.05 vs. N-IMC gel for each category. Almost all IMC was in the solid state in the IMC transdermal formulations (ratio, solid form: solution form = 98.6: 1.4), and the drug homogeneity (dispersity, standard error) in the solid nanoparticles (SNPs)-based transdermal formulations was higher than in the microparticles-based transdermal formulations. The zeta potentials of the IMC transdermal formulations with or without l-menthol were similar, and the IMC contents in the microparticles-and SNPs-based transdermal formulations did not change for 1 month.

**Figure 3:**
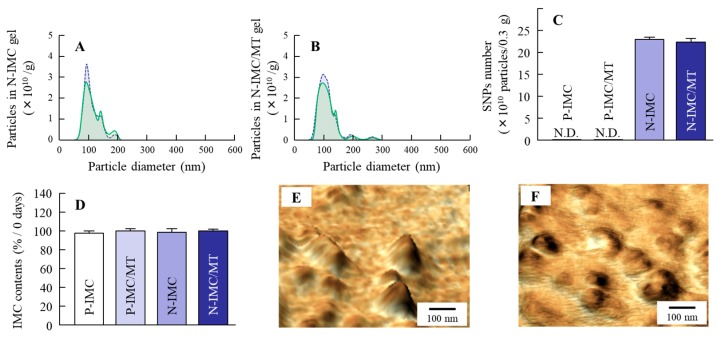
Particle analysis of N-IMC and N-IMC/MT gels 1 month after preparation. (**A** and **B**); Particle size frequencies in N-IMC (**A**) and N-IMC/MT (**B**) gels immediately (blue) and 1 month (green) after preparation. (**C**); the number of SNPs in IMC transdermal formulations. (**D**); the changes in IMC content in the transdermal formulations. **E** and **F**; AFM images of SNPs in N-IMC (**E**) and N-IMC/MT (**F**) gels. *n* = 6–10. The IMC particle size of SNPs-based transdermal formulations were not changed 1 month after preparation, and the particle sizes in the N-IMC and N-IMC/MT gels were 109.8 ± 6.9 nm and 115.3 ± 7.1 nm, respectively.

It is important to evaluate any changes in particle characterization by combination with l-menthol and by long-term storage. Therefore, this study compared the particle size frequencies, solubility, zeta potential and stability of IMC transdermal formulations with or without l-menthol (Figure 1 and Figure 2), and investigated the changes in particle size frequencies and IMC content of the IMC transdermal formulations 30 days after preparation (Figure 3). The bead mill treatment led to a decrease in the particle size of IMC to a mean particle size of 104.6 ± 6.4 nm in the NANOSIGHT LM10 (Figure 1E). Moreover, the particle characterizations were not affected by a combination with l-menthol (Figure 1F). The particles were uniformly dispersed in the transdermal formulations containing IMC SNPs with (N-IMC/MT gel) or without l-menthol (N-IMC gel), and no drug degradation was observed after 30 days (Figure 2A and Figure 3D). The zeta potentials of the IMC transdermal formulations were approximately 18–21 mV (Figure 2C). Although, solubility was enhanced by the bead mill treatment, the ratio of solid particles to the dissolved form in both the N-IMC and N-IMC/MT gels was 98.6: 1.4 (Figure 2B). In addition, the nanoparticles in the N-IMC and N-IMC/MT gels did not aggregate or degrade for 30 days (mean particle size: N-IMC gel 109.8 ± 6.9 nm; N-IMC/MT gel 115.3 ± 7.1 nm), and the nanoparticle form in the N-IMC and N-IMC/MT gels was similar (Figure 3).

### 2.2. Behavior of IMC Release from IMC Transdermal Formulations with or without l-Menthol

**Figure 4:**
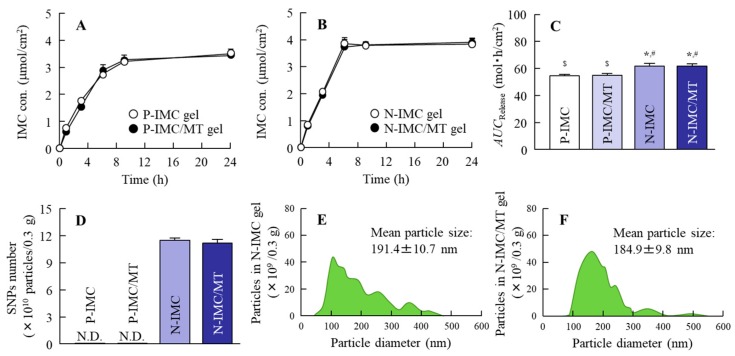
IMC release from the transdermal formulations through 20 µm pore membranes. (**A**); the drug release from the P-IMC and P-IMC/MT gels through the membranes. (**B**); the drug release from the N-IMC and N-IMC/MT gels through the membranes. (**C**); the area under the IMC concentration-time curve of drug release (*AUC*_Release_) for the IMC transdermal formulations. (**D**); the number of IMC SNPs released from the transdermal formulations 24 h after application. (**E**) and (**F**); the size frequencies of IMC released from the N-IMC (**E**) and N-IMC/MT (**F**) gels 24 h after application. These samples were collected in the reservoir chamber. *n* = 7. * *p* < 0.05 vs. P-IMC gel for each category. ^#^ *p* < 0.05 vs. P-IMC/MT gel for each category. ^$^ *p* < 0.05 vs. N-IMC gel for each category. The combination with l-menthol did not affect drug release from the IMC transdermal formulations, and the IMC released from the N-IMC and N-IMC/MT gels was in the SNPs state (mean particle size, N-IMC gel 191.4 nm, N-IMC/MT gel 184.9 nm).

Figure 4 shows the drug release from the IMC transdermal formulations with or without l-menthol. The amount of drug that penetrated through a 20 µm pore membrane from the N-IMC gel was higher than the transdermal formulation (P-IMC gel) containing IMC solid microparticles (powder), and no difference was observed between the corresponding formulations with (P-IMC/MT gel) or without (P-IMC gel) l-menthol (Figure 4A–C). The drug release rate constant (*k*_r_) of P-IMC and P-IMC/MT gels were 0.23 ± 0.01/h and 0.23 ± 0.02/h, respectively. In addition, the *k*_r_ of N-IMC/MT gel (0.26 ± 0.02/h) was similar to N-IMC gel (*k*_r_, 0.27 ± 0.01/h). Although no particles appeared in the reservoir chamber treated with P-IMC and P-IMC/MT gels, approximately 80–500 nm IMC SNPs were detected in the reservoir chamber treated with N-IMC and N-IMC/MT gels (Figure 4D–F).

### 2.3. IMC Penetration into the Rat Skin when IMC Transdermal Formulations with or without l-Menthol Were Applied

**Figure 5:**
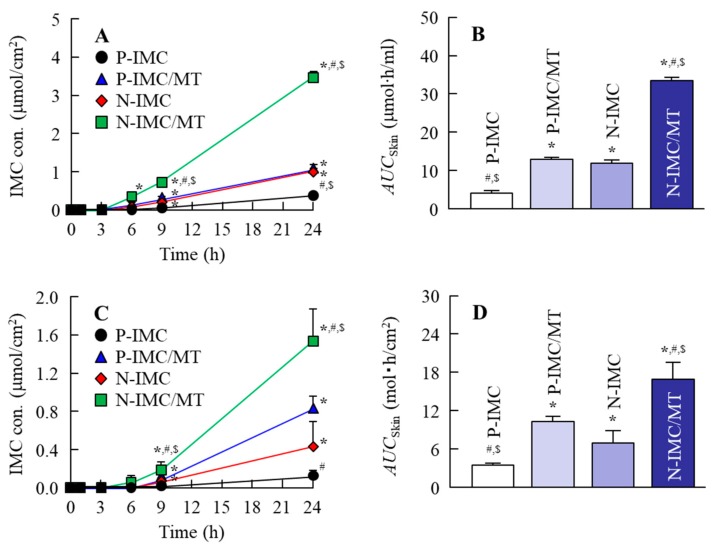
In vitro skin penetration of IMC transdermal formulations. (**A** and **B**); penetration (**A**) and *AUC*_Skin_ (**B**) for the IMC transdermal formulations through rat skin under normal conditions (37 °C). (**C** and **D**); penetration (**C**) and *AUC*_Skin_ (**D**) for the IMC transdermal formulations through rat skin under low temperature conditions (4 °C). *n* = 6–8. * *p* < 0.05 vs. P-IMC gel for each category. ^#^ *p* < 0.05 versus P-IMC/MT gel for each category. ^$^ *p* < 0.05 vs. N-IMC gel for each category. The combination with l-menthol enhanced the skin penetration of IMC from both the microparticles-and SNPs-based transdermal formulations, and the *AUC*_skin_ values for the N-IMC and N-IMC/MT gels were 2.9 and 2.6-fold higher in comparison with the corresponding microparticles-based transdermal formulations, respectively. Although skin penetration from the P-IMC/MT gel did not differ significantly between 4 °C and 37 °C, the skin penetration from the N-IMC/MT gel was remarkably attenuated at 4 °C.

**Figure 6:**
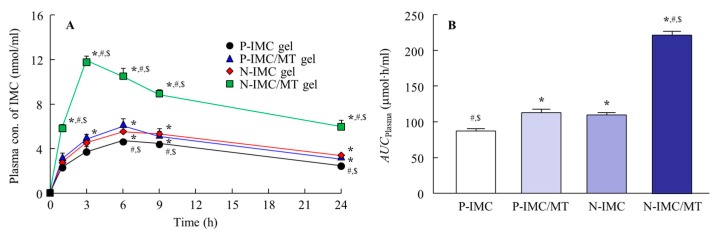
The percutaneous absorption of IMC transdermal formulations. (**A**) Transdermal penetration for the IMC transdermal formulations. (**B**) *AUC*_Plasma_ values for the IMC transdermal formulations. *n* = 6–7. * *p* < 0.05 vs. P-IMC gel for each category. ^#^ *p* < 0.05 vs. P-IMC/MT gel for each category. ^$^ *p* < 0.05 vs. N-IMC gel for each category. The difference between the N-IMC and N-IMC/MT gels was significantly higher than that of the corresponding microparticles-based transdermal formulations, and the *AUC*_Plasma_ for the N-IMC/MT gel was 2-fold higher than for the N-IMC gel.

Figure 5A,B show the profiles of skin penetration from IMC transdermal formulations with or without l-menthol at 37 °C (normal conditions), and Table 1 summarizes the pharmacokinetic parameters calculated from the data in Figure 5A. Skin penetration and the penetration rate (*J*_c_) for the N-IMC gel were significantly higher than the P-IMC gel, and the area under the IMC concentration-time curve of drug release of skin penetration (*AUC*_Skin_) was similar to the P-IMC/MT gel. On the other hand, skin penetration and *J*_c_ were remarkably enhanced by the combination of IMC SNPs and l-menthol, with *AUC*_Skin_ and *J*_c_ values for the N-IMC/MT gel 2.8-and 3.8-fold higher than the N-IMC gel, respectively. In contrast to the results for drug release (Figure 4), no IMC SNPs were detected in the reservoir chamber treated with any of the four transdermal formulations by NANOSIGHT LM10. The authors then measured the effect of the energy-dependent uptake on the skin penetration of N-IMC/MT gel using low temperature (4 °C) conditions [22], where all energy-dependent endocytosis was prevented (Figure 5C,D). For the P-IMC and P-IMC/MT gels, no significant difference was observed in the corresponding *AUC*_Skin_ values between the 4 °C and 37 °C conditions. In contrast, the skin penetration of the IMC SNPs was attenuated under low temperature (4 °C) conditions as compared with 37 °C conditions. However, the enhancing effect on skin penetration by l-menthol was maintained, and the *AUC*_Skin_ for the P-IMC/MT gel was 2.5-fold higher than the P-IMC gel. Figure 6 shows the percutaneous absorption for IMC transdermal formulations with or without l-menthol, and Table 2 summarizes the pharmacokinetic parameters analyzed from the data in Figure 6. The area under the IMC concentration-time curve of the drug release of percutaneous absorption (*AUC*_Plasma_) and apparent absorption rate constant (*k*_a_) values were also enhanced by pulverization and the addition of l-menthol in the in vivo study, and the parameters for the N-IMC gel were significantly higher than for the P-IMC gel. In addition, the *AUC*_Plasma_ and *k*_a_ values for the N-IMC/MT gel were 2.0-and 2.4-fold higher than the N-IMC gel.

## 3. Discussion

The IMC transdermal formulations are useful as therapy for inflammation and pain, since TDD avoids serious adverse effects, such as gastrointestinal injury. However, the SC plays a role as a biologic protector against the entrance of foreign substances into the body, and it is difficult to deliver drugs into the blood through the skin. A variety of strategies are available to overcome low skin permeability, and pro-drugs, iontophoresis, magnetophoresis, needleless injection, sonophoresis, microporation, electroporation, colloidal formulations, and chemical permeation enhancers have been introduced in previous studies [23,24,25,26]. In particular, the reduction of drug particle sizes to the nano-order prompts a dramatic increase in cellular uptake and skin penetration [12,13,14]. Our previous studies using NSAIDs (ketoprofen) also showed that the percutaneous absorption of drug SNPs-based transdermal formulations was higher than traditional formulations [15,16,17]. This study aimed to increase the skin penetration of drug SNPs-based transdermal formulations, and investigated whether the combination with l-menthol can enhance the skin penetration and drug absorption of the N-IMC gel.

First, SNPs-based TDD of IMC with l-menthol (N-IMC/MT gel) was prepared, and its stability was evaluated. Almost all the IMC was present in a solid condition in the transdermal formulations, and the mean particle sizes in the N-IMC and N-IMC/MT gels was 104.6 ± 6.4 nm and 109.1 ± 6.9 nm, respectively (Figure 1C–H). Moreover, the SNPs-based transdermal formulations with or without l-menthol showed high drug homogeneity (Figure 2A), and the zeta potentials of the SNPs-based transdermal formulations with or without l-menthol were approximately −10.7 (Figure 2C). In addition, the particle size, shape, and drug contents of both the N-IMC and N-IMC/MT gels did not change after storage for 1 month (Figure 3D). It has previously been reported that SNPs with diameters in the range of 60–100 nm were optimal for the cellular uptake process and skin penetration [27,28,29]. Based on the results showing that acombination with l-menthol does not affect the stability of SNPs-based transdermal formulations, and that almost half of the SNPs in the SNPs-based transdermal formulations (N-IMC and N-IMC/MT gels) designed in this study are suitable for a transdermal delivery system (i.e., <100 nm), the formulations may be suitable candidates for therapeutic use.

This study used the Franz diffusion cell to demonstrate the release of IMC particles from the IMC transdermal formulations. SNPs were observed in the IMC released from both the N-IMC and N-IMC/MT gels with similar IMC amounts, particle size frequencies, and particle numbers (Figure 4). These results show that IMC is released from the N-IMC and N-IMC/MT gels as both of SNPs and in a dissolved form, and that the combination with l-menthol does not affect drug release. 

**Figure 7:**
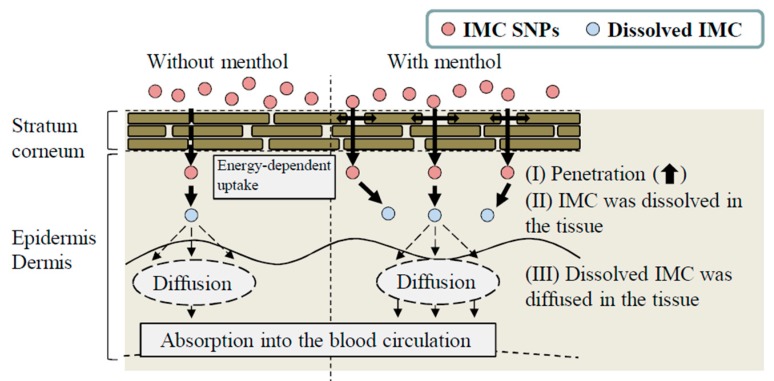
The mechanism for the percutaneous absorption process by the combination of IMC SNPs and l-menthol.

Next, the effect of l-menthol on the skin penetration of the SNPs-based transdermal formulations was demonstrated. The skin comprises outer (epidermis), middle (dermis), and inner (subcutaneous tissue) layers, and the epidermis is divided into the SC (non-viable epidermis, which is hydrophobic) and the viable epidermis (hydrophilic). The thickness of the dermis is approximately 0.5–3 mm, and contains nerve endings, sweat glands, lymph vessels, and blood vessels. The subcutaneous tissue in the innermost layer relates to physical protection, nutritional support and temperature regulation [30,31,32]. In terms of the skin penetration of drugs, it is known that it is difficult for hydrophilic drugs to penetrate through the SC, since the SC is hydrophobic. Although the SC acts as a barrier for hydrophilic drugs, small amounts of hydrophilic drugs can penetrate through the SC. However, the penetrating hydrophilic drugs show limited ability to permeate through the hydrophilic viable epidermis [30,31,32]. Therefore, TDDs require careful design to allow for the skin penetration of drugs. For TDDs containing SNPs, our previous studies using ketoprofen, tranilast, indomethacin, ibuprofen and raloxifene demonstrated that SNPs with particle sizes under 100 nm penetrated into the SC [15,16,17,18,19,33]. Furthermore, these SNPs permeate by energy-dependent endocytosis and the drugs that are dissolved in this process are delivered into the systemic circulation [15]. The particles in the N-IMC gel were approximately 50–200 nm in size, and skin penetration of IMC was observed by application of the N-IMC gel, and the skin penetration of N-IMC gel was higher in comparison with the P-IMC gel consisting of IMC microparticles (Figure 5A,B). On the other hand, the skin penetration was attenuated under low temperature (4 °C) conditions [22] at which the function of all energy-dependent uptake, including endocytosis, is inhibited in cells (Figure 5C,D). In addition, no IMC nanoparticles were detected in the reservoir chamber treated with N-IMC or N-IMC/MT gels. These results using the N-IMC gel support previous studies using ketoprofen, tranilast, indomethacin, ibuprofen and raloxifene [15,16,17,18,19,33]. On the other hand, the combination with l-menthol enhanced the IMC skin penetration rate (*J*_c_ and *k*_a_) of the N-IMC/MT gel (Table 1 and Table 2), and the *AUC*_Skin_ of the N-IMC/MT gel was 2.5-fold higher than the N-IMC gel (Figure 5D). Moreover, the *AUC*_Plasma_ of the N-IMC/MT gel was also higher than N-IMC, and the difference between the N-IMC and N-IMC/MT gels was significantly higher than that between the corresponding P-IMC-based transdermal formulations (Figure 5). Kaplun-Frischoff and Touitou [21] reported that l-menthol spreads the cell gap in SC, and enhances skin absorption via alterations in the barrier properties of the SC. In addition, the skin penetration of N-IMC/MT gel was higher than the N-IMC gel, although skin penetration was attenuated under the low temperature conditions (Figure 5C,D). Taken together, it was hypothesized that l-menthol decreases the barrier function of the SC, and this alteration allows easy penetration of the N-IMC gel to penetrate more easily through the SC. The penetrated IMC SNPs may be taken up into the cells (viable epidermis) by energy-dependent endocytosis, and may shift to the dermis and subcutaneous tissue layers. During this process, the N-IMC gel is dissolved, and delivered into the blood circulation, resulting in the increase in percutaneous absorption (Figure 7).

Further studies are necessary to clarify the dissolution mechanism of IMC SNPs in the percutaneous absorption process. In addition, it is necessary to elucidate the kind of energy-dependent endocytosis that is involved in skin penetration during IMC SNPs-based transdermal penetration. Therefore, the authors are investigating the effect of energy-dependent endocytosis on the dissolution of the N-IMC gel using endocytosis selected inhibitors, such as nystatin (caveolae-mediated endocytosis inhibitor), dynasore (clathrin-mediated endocytosis inhibitor), rottlerin (macropinocytosis inhibitor), and cytochalasin D (phagocytosis inhibitor) [34,35,36].

## 4. Materials and Methods 

### 4.1. Animals and Reagents

The IMC and l-menthol were obtained from Wako Pure Chemical Industries, Ltd. (Osaka, Japan). Further, 2-hydroxypropyl-β-cyclodextrin (HPβCD) and methylcellulose (MC) were purchased from Nihon Shokuhin Kako Co., Ltd. (Tokyo, Japan) and Shin-Etsu Chemical Co., Ltd. (Tokyo, Japan), respectively. The membranes (polycarbonate track etched membrane) for the evaluation of drug release (pore size 20 µm) were obtained from GVS Japan (Tokyo, Japan), and carboxypolymethylene (Carbopol^®^ 934) was provided by Serva (Heidelberg, Germany). The seven-week-old Wistar rats (Kiwa Laboratory Animals Co., Ltd., Wakayama, Japan) were used in this study, and experiments were carried out in accordance with the Guidelines for the Care and Use of Laboratory Animals of both the Japanese Pharmacological Society and Kindai University (identification code KAPS-25-002, 1 April 2013).

### 4.2. Design of IMC SNPs-based Transdermal Formulations with or without l-Menthol

Drug SNPs were prepared according to our previous reports [15,17,19]. Briefly, IMC powder was dispersed in distilled water containing 5% HPβCD and 0.5% MC was selected as additives to prevent aggregation and enhance the mill power, respectively for 3 h at 22 °C. The dispersions were treated by the bead mill method using Bead Smash 12 (Wakenyaku Co. Ltd., Kyoto, Japan) for 30 times (3000 rpm, 30 s, 4 °C), and the pH of each formulations are adjusted to 7. In preparation of IMC transdermal formulations containing 2% menthol, the menthol was added to the milled IMC dispersion, then stirred and sonicated for 1 h. After that, the IMC dispersions with or without menthol were gelled by carbopol dissolved in distilled water at 22 °C (with menthol, N-IMC/MT gel; without menthol, N-IMC gel). The transdermal formulations containing IMC microparticles were prepared by the following method. The 1% IMC powder was mixed with 5% HPβCD 0.5% MC, and/or 2% menthol, and dispersed in distilled water, and stirred for 1 h. The dispersions were gelled by carbopol dissolved in distilled water at 22 °C (with menthol, P-IMC/MT gel; without menthol, P-IMC gel). The P-IMC and P-IMC/MC gels were used to compare the IMC SNPs-based transdermal formulations. The viscosity in these P-IMC, P-IMC/MT, N-IMC and P-IMC/MC gels were similar, and the values were approximately 12–16 Pa∙s.

### 4.3. Particle Characteristics of the IMC Transdermal Formulations

The particle size and number of IMC SNPs were measured by a NANOSIGHT LM10 (QuantumDesign Japan, Tokyo, Japan) as follows: Viscosity 1.27 mPa∙s; wavelength 405 nm (blue); time 60 s. The atomic force microscope (AFM) images of the SNPs were evaluated using a SPM (scanning probe microscope)-9700 (Shimadzu Corp., Kyoto, Japan). The zeta potential of the IMC particles in the gels were measured using a Zeta Potential Meter Model 502 (Nihon Rufuto Co., Ltd., Tokyo, Japan). In this study, the IMC transdermal formulations were divided into 10 parts, and the dispersity of each part was analyzed for IMC content to evaluate the homogeneity in the IMC transdermal formulations.

### 4.4. Measurement of IMC

The IMC concentrations were measured by a Shimadzu LC-20AT system equipped with a column oven CTO-20A (Shimadzu Corp., Kyoto, Japan). Propyl p-hydroxybenzoate was selected as the internal standard, and an Inertsil^®^ODS-3 column (3 µm) was used (GL Science Co. Inc., Tokyo, Japan). Other conditions were as follows: Column temperature, 35 °C; flow rate, 0.25 mL/min; mobile phase, acetonitrile/50 mM acetic acid (40/60, *v*/*v*); wavelength for detection, 254 nm. 

### 4.5. Evaluation of Drug Release from IMC Transdermal Formulations

A Franz diffusion cell with 20 µm pore membranes was used to measure drug release from IMC transdermal formulations according to the previous studies [15,17,19]. The formulation samples (0.3 g) were applied on the membrane surface in the donor compartment, and the reservoir chamber was filled by 0.85% NaCl-10 mM phosphate buffer (pH 7.4). The diffusion cells were thermoregulated in a water bath at 37 °C for 24 h. One hundred microliter aliquots of sample solution were withdrawn from the reservoir chamber (reservoir volume 12.2 mL). The area under the IMC concentration-time curve (*AUC*_Release_) was calculated by the trapezoidal rule up to the last measurement point (24 h), and the drug release rate constant (*k*r/h) was analyzed according to Equation (1):

(1) $$C_{t}=C_{\infty }\cdot \left(1-e^{-k_{r}\cdot t}\right)$$

where *t* is time (0–24 h), and *C*_∞_ and *C*_t_ are the IMC concentration at time ∞ and *t*, respectively.

### 4.6. Evaluation of Skin Penetration of IMC Transdermal Formulations

A Franz diffusion cell fitted with abdominal rat skin instead of a filter was used according to our previous study [15]. On the day before the experiment, the hair on the abdominal area of 7 week-old Wistar rats was carefully removed with an electric clipper and electric razor. The following day, pieces (3 cm × 3 cm area) of full-thickness abdominal skin were excised from the rats, and the adherent fat and other visceral debris were removed from the undersurface. The dermal side of the full-thickness skin (approximately 1 mm) was soaked in a buffer (0.85% NaCl-10 mM phosphate buffer, pH 7.4) for 12 h at 4 °C to equilibrate the skin. The transdermal formulations containing 0.3 g IMC were spread uniformly over the abdominal rat skin (effective area of the skin, *A*, 2 cm^2^), and the samples (100 µL) were withdrawn from the reservoir chamber filled the 0.85% NaCl-10 mM phosphate buffer (pH 7.4) over time. The samples were analyzed for drug concentration, particle size and number as described above. In addition, pharmacokinetic parameters (the penetration rate, *J*_c_; the skin/preparation partition coefficient, *K*_m_; the penetration coefficient through the skin, *K*_p_; the diffusion constant within the skin, *D*; *t*_lag_, lag time; the thickness of the skin, *δ*(0.071 cm); the amount of IMC (*C*_IMC_) in the reservoir solution at time *t*, *Q*_t_) were calculated according to Equations (2)–(4):

(2) $$t_{\mathrm{lag}}=\frac{\delta^{2}}{6D}$$

(3) $$J_{c}=\frac{K_{m}\cdot D\cdot C_{\mathrm{IND}}}{\delta }=K_{p}\cdot C_{\mathrm{IND}}$$

(4) $$Q_{t}=J_{c}\cdot A\cdot \left(t-t_{\mathrm{lag}}\right)$$

The *AUC*_Skin_ was estimated by the trapezoidal rule up to the last measurement point (24 h).

### 4.7. Evaluation of Percutaneous Absorption from IMC Transdermal Formulations

The abdominal skin of rats was shaved with an electric-clipper and razor, and 0.3 g of a IMC transdermal formulation was applied uniformly over the effective area (2 cm^2^). After application, blood (200 µL) was collected from the right jugular vein for the measurement of plasma IMC concentrations by the HPLC method described above. The *AUC*_Plasma_ was analyzed by the trapezoidal rule up to the last measurement point (24 h), and the pharmacokinetics parameters (the elimination rate constant, *k*_e_; the distribution volume, *V*_d_; the apparent absorption rate constant, *k*_a_; *C*_IMC_, the IMC concentration; *D*, the dose of IMC; time, *t* (0–24 h); the lag time (h), *t*_lag_; the fraction of IMC absorbed, *F*) were calculated according to Equations (5) and (6):

(5) $$C_{\mathrm{IND}}=C_{0}\cdot e^{-K_{c}\cdot t}$$

(6) $$C_{\mathrm{IND}}=\frac{K_{a}\cdot F\cdot D}{V_{d}\cdot \left(K_{a}-K_{e}\right)}\cdot \left(e^{-K_{e}\cdot \left(t-t_{\mathrm{lag}}\right)}+e^{-K_{a}\cdot \left(t-t_{\mathrm{lag}}\right)}\right)$$

The *k*_e_ and *V*_d_ were analyzed by Equation (5), and the IMC concentration in the plasma after a single injection of 0.3 mL of IMC solution (200 µg/kg) into the femoral vein was used. The *C*_0_ (initial concentration of IMC in the plasma), *k*_e_, and *V*_d_ were 2.68 ± 0.13 µg/mL, 0.05 ± 0.07 h^−1^, and 52.1 ± 1.98 mL/kg, respectively (*n* = 5). The *k*_a_ and *F* in the percutaneous absorption experiment were estimated by Equation (6).

### 4.8. Characterization of IMC

The IMC with or without bead mill treatment were lyophilized, and the morphology was characterized by using a powder X-ray diffraction (XRD) method. The XRD analysis was performed using a Mini Flex II (Rigaku, Co., Tokyo, Japan) instrument with a Cu-Kα target. The x-rays were done at 30 kV and 15 mA. The data were obtained from 5° to 90° diffraction angles with a scanning rate of 10°/min. The morphology of IMC was no different with or without bead mill treatment (supplemental data).

### 4.9. Statistical Analysis

The data in SALD-7100 are expressed as the mean ± standard error of the mean. The student’s *t*-test and Dunnett’s multiple comparison were used for analysis of two and multiple groups, respectively. A minimum *p* value of 0.05 (*p* < 0.05) was chosen as the significance level.

## 5. Conclusions

This study designed transdermal formulations containing IMC SNPs and l-menthol, and the IMC particle size of SNPs-based transdermal formulations were approximately 50–200 nm, and the IMC SNPs remained 1 month after preparation. Moreover, it was found that the skin penetration of 50–200 nm IMC was low, although, the combination with l-menthol enhanced the skin penetration of IMC SNPs with 50–200 nm particles. It is possible that the barrier function of the SC decreased by l-menthol, and that this change led to the enhanced permeation of IMC SNPs. Moreover, the energy-dependent endocytosis in the skin tissue is related to the skin penetration of IMC SNPs (Figure 7). These findings suggest that solid nanoparticles in combination with l-menthol are useful for transdermal delivery of IMC, and the SNPs-based formulation may represent a novel transdermal therapeutic system for the management of inflammation.

## Figures and Tables

**Table 1:** Pharmacokinetic analysis of IMC transdermal formulations in in vitro skin penetration.

Formulation	*J_c_* (nmol/cm^2^/h)	*K*_p_ (×10^−4^ cm/h)	*K_m_* (×10^−2^)	*τ* (h)	*D* (×10^−4^ cm^2^/h)
P-IMC gel	18.6 ± 1.8 ^#,$^	0.66 ± 0.06 ^#,$^	0.92 ± 0.09 ^#,$^	1.65 ± 0.03 ^#,$^	5.10 ± 0.11^#,$^
P-IMC/MT gel	43.1 ± 3.1 *	1.52 ± 0.11 *	2.68 ± 0.28 *	2.03 ± 0.07 *	4.10 ± 0.11 *
N-IMC gel	41.6 ± 4.6 *	1.49 ± 0.17 *	2.51 ± 0.22 *	2.00 ± 0.06 *	4.20 ± 0.12 *
N-IMC/MT gel	158.1 ± 4.2 *^,#,$^	5.65 ± 0.15 *^,#,$^	10.8 ± 0.29 *^,#,$^	2.27 ± 0.02 *^,#,$^	3.71 ± 0.04 *^,#,$^

The experiments were performed at 37 °C. *n* = 6–8. * *p* < 0.05 vs. P-IMC gel for each category. ^#^ *p* < 0.05 vs. P-IMC gel for each category. ^$^ *p* < 0.05 vs. N-IMC gel for each category.

**Table 2:** Pharmacokinetic analysis of percutaneous absorption of the IMC transdermal formulations.

Parameter	P-IMC Gel	P-IMC/MT Gel	N-IMC Gel	N-IMC/MT Gel
*k_a_* (h^−1^)	0.18 ± 0.06 ^#,$^	0.37 ± 0.05 *	0.34 ± 0.04 *	0.83 ± 0.03 *^,#,^^$^
*F* (×10^−3^)	0.14 ± 0.03	0.16 ± 0.02	0.15 ± 0.02	0.21 ± 0.01 *^,#,^^$^

The elimination rate constant (*k*_e_) was 0.05 ± 0.07 h^−1^. *n* = 6–7. * *p* < 0.05 vs. P-IMC gel for each category. ^#^ *p* < 0.05 vs. P-IMC/MT gel for each category. ^$^ *p* < 0.05 vs. N-IMC gel for each category.

## Abbreviations

AFM	atomic force microscope
*AUC*	area under the indomethacin concentration-time curve
carbopol	Carbopol^®^ 934
COX	cyclooxygenase
*D*	diffusion constant within the skin
HPβCD	2-hydroxypropyl-β-cyclodextrin
IMC	indomethacin
P-IMC gel	transdermal formulations containing indomethacin solid microparticles
N-IMC gel	transdermal formulations containing indomethacin solid nanoparticles
*J*_c_	penetration rate
*k*_a_	apparent absorption rate constant
*k*_e_	elimination rate constant
*K*_m_	skin/preparation partition coefficient
*K*_p_	penetration coefficient through the skin
*k*_r_	drug release rate constant
MC	methylcellulose
P-IMC/MT gel	transdermal formulations containing indomethacin solid microparticles and l-menthol
N-IMC/MT gel	transdermal formulations containing indomethacin solid nanoparticles and l-menthol
NSAIDs	non-steroidal anti-inflammatory drug
SC	stratum corneum
SNPs	solid nanoparticles
TDD	transdermal drug delivery
*t*_lag_	lag time
XRD	powder X-ray diffraction

## References

[1] Sostres C., Gargallo C.J., Arroyo M.T., Lanas A. (2010). Adverse effects of non-steroidal anti-inflammatory drugs (NSAIDs, aspirin and coxibs) on upper gastrointestinal tract. Best Pract. Res. Clin. Gastroenterol..

[2] Bateman D.N. (2012). Non-steroidal anti-inflammatory drugs. Medicine (Baltimore).

[3] Kim S.J., Flach A.J., Jampol L.M. (2010). Nonsteroidal anti-inflammatory drugs in ophthalmology. Surv. Ophthalmol..

[4] Nagai N., Fukuhata T., Ito Y., Usui S., Hirano K. (2009). Involvement of interleukin 18 in indomethacin-induced lesions of the gastric mucosa in adjuvant-induced arthritis rat. Toxicology.

[5] Kato S., Takeuchi K. (2002). Alteration of gastric ulcerogenic and healing responses in rats with adjuvant-induced arthritis. Jpn. J. Pharmacol..

[6] Ren C., Fang L., Ling L., Wang Q., Liu S., Zhao L., He Z. (2009). Design and in vivo evaluation of an indapamide transdermal patch. Int. J. Pharm..

[7] Shinkai N., Korenaga K., Mizu H., Yamauchi H. (2008). Intra-articular penetration of ketoprofen and analgesic effects after topical patch application in rats. J. Control. Release.

[8] So J.W., Park H.H., Lee S.S., Kim D.C., Shin S.C., Cho C.W. (2009). Effect of microneedle on the pharmacokinetics of ketoprofen from its transdermal formulations. Drug Deliv..

[9] Williams A.C., Barry B.W. (2004). Penetration enhancers. Adv. Drug Deliv. Rev..

[10] Djordjevic L., Primorac M., Stupar M. (2005). In vitro release of diclofenac diethylamine from caprylocaproyl macrogolglycerides based microemulsions. Int. J. Pharm..

[11] Podlogar F., Bester Rogac M., Gasperlin M. (2005). The effect of internal structure of selected water—Tween 40^®^—Imwitor 308^®^—IPM microemulsions on ketoprofene release. Int. J. Pharm..

[12] Soma D., Attari Z., Reddy M.S., Damodaram A., Koteshwara K.B.G. (2017). Solid lipid nanoparticles of irbesartan: Preparation, characterization, optimization and pharmacokinetic studies. Braz. J. Pharm. Sci..

[13] Wissing A.S., Müller R.H. (2003). Cosmetic applications for solid lipid nanoparticles (SLN). Int. J. Pharm..

[14] Montenegro L., Lai F., Offera A., Sarpietro M.G., Micicche L., Maccioni A.M., Valenti D., Fadda A.M. (2016). From nanoemulsions to nanostructured lipid carriers: A relevant development in dermal delivery of drugs and cosmetics. J. Drug Deliv. Sci. Technol..

[15] Nagai N., Ogata F., Ishii M., Fukuoka Y., Otake H., Nakazawa Y., Kawasaki N. (2018). Involvement of Endocytosis in the Transdermal Penetration Mechanism of Ketoprofen Nanoparticles. Int. J. Mol. Sci..

[16] Nagai N., Iwamae A., Tanimoto S., Yoshioka C., Ito Y. (2015). Pharmacokinetics and Antiinflammatory Effect of a Novel Gel System Containing Ketoprofen Solid Nanoparticles. Biol. Pharm. Bull..

[17] Nagai N., Ito Y. (2014). Therapeutic Effects of Gel Ointments containing Tranilast Nanoparticles on Paw Edema in Adjuvant-Induced Arthritis Rats. Biol. Pharm. Bull..

[18] Nagai N., Yoshioka C., Ito Y. (2015). Topical Therapies for Rheumatoid Arthritis by Gel Ointments containing Indomethacin Nanoparticles in Adjuvant-Induced Arthritis Rat. J. Oleo Sci..

[19] Nagai N., Tanino T., Ito Y. (2016). Pharmacokinetic Studies of Gel System Containing Ibuprofen Solid Nanoparticles. J. Oleo Sci..

[20] Sinha V.R., Kaur M.P. (2000). Permeation enhancers for transdermal drug delivery. Drug Dev. Ind. Pharm..

[21] Kaplun-Frischoff Y., Touitou J. (1997). Testosterone skin permeation enhancement by menthol through formation of eutectic with drug and interaction with skin lipids. Pharm. Sci..

[22] He Z., Liu K., Manaloto E., Casey A., Cribaro G.P., Byrne H.J., Tian F., Barcia C., Conway G.E., Cullen P.J. (2018). Cold Atmospheric Plasma Induces ATP-Dependent Endocytosis of Nanoparticles and Synergistic U373MG Cancer Cell Death. Sci. Rep..

[23] Rehman K., Zulfakar M.H. (2013). Recent advances in gel technologies for topical and transdermal drug delivery. Drug Dev. Ind. Pharm..

[24] Brambilla D., Luciani P., Leroux J. (2014). Breakthrough discoveries in drug delivery technologies: The next 30 years. J. Control. Release.

[25] Han T., Das D.B. (2015). Potential of combined ultrasound and microneedles for enhanced transdermal drug permeation: A review. Eur. J. Pharm. Biopharm..

[26] Ahad A., Aqil M., Kohli K., Sultana Y., Mujeeb M., Ali A. (2010). Transdermal drug delivery: The inherent challenges and technological advancements. Asian J. Pharm. Sci..

[27] Gao H., Shi W., Freund L.B. (2005). Mechanics of receptor-mediated endocytosis. Proc. Natl. Acad. Sci. USA.

[28] Zhang S., Gao H., Bao G. (2015). Physical Principles of Nanoparticle Cellular Endocytosis. ACS Nano.

[29] Chithrani B.D., Chan W.C. (2007). Elucidating the mechanism of cellular uptake and removal of protein-coated gold nanoparticles of different sizes and shapes. Nano Lett..

[30] Tanwar H., Sachdeva R. (2016). Transdermal drug delivery system: A review. IJPSR.

[31] Mbah C.J., Uzor P.F., Omeje E.O. (2011). Perspective on transdermal drug delivery. J. Chem. Pharm. Res..

[32] Bartosova L., Bajgar J. (2012). Transdermal drug delivery in vitro using diffusion cells. Curr. Med. Chem..

[33] Nagai N., Ogata F., Otake H., Nakazawa Y., Kawasaki N. (2018). Design of a transdermal formulation containing raloxifene nanoparticles for osteoporosis treatment. Int. J. Nanomed..

[34] Hufnagel H., Hakim P., Lima A., Hollfelder F. (2009). Fluid phase endocytosis contributes to transfection of DNA by PEI-25. Mol. Ther..

[35] Malomouzh A.I., Mukhitov A.R., Proskurina S.E., Vyskocil F., Nikolsky E.E. (2014). The effect of dynasore, a blocker of dynamin-dependent endocytosis, on spontaneous quantal and non-quantal release of acetylcholine in murine neuromuscular junctions. Dokl. Biol. Sci..

[36] Mäger I., Langel K., Lehto T., Eiríksdóttir E., Langel U. (2012). The role of endocytosis on the uptake kinetics of luciferin-conjugated cell-penetrating peptides. Biochim. Biophys. Acta Biomembr..

